# A qualitative study of the value of simulation-based training for nursing students in primary care

**DOI:** 10.1186/s12912-024-01886-0

**Published:** 2024-05-06

**Authors:** Lucy Bray, Doris Østergaard

**Affiliations:** 1 Copenhagen Academy for Medical Education and Simulation, Centre for HR and Education, Copenhagen, Denmark; 2 Department of Clinical Medicine, University of Copenhagen, Copenhagen, Denmark

**Keywords:** Nursing education, Primary care, Simulation-based training

## Abstract

**Background:**

Clinical placement is an essential component of nursing education, providing students with the opportunity to apply theoretical knowledge to practice. However, challenges such as lack of supervision and passive involvement in tasks can hinder the learning experience. Supplementing clinical placement with simulation-based training (SBT) has been explored as a potential solution, though this approach has been underexplored within primary care. This study aimed to explore the educational value of a supplemental SBT course for nursing students during primary care placement, as well as the adaption of this approach to a primary care setting.

**Methods:**

A qualitative descriptive study was conducted at a medical education and simulation academy in Denmark. Sixth-semester nursing students on placement in primary care were invited to participate. The intervention consisted of a three-day simulation course covering core nursing competencies and common clinical conditions encountered within primary care. Simulation adopted a standardised patient approach. Data was collected using focus group interviews, which was analysed using inductive thematic analysis.

**Results:**

Thirty-one nursing students participated in the study. Seven themes emerged from the analysis, including perceptions, educational value, simulation adjustments to primary care, educators’ competencies, learning needs within primary care, challenges of clinical placement and career guidance. Generally, participants perceived the intervention positively, appreciating its relevance to their clinical placement and its educational impact in this context. Participants also provided insights into the adaptation of SBT to a primary care setting, as well as nursing students’ learning needs within this context.

**Conclusion:**

The findings indicate that the intervention had a positive impact on participant competencies within this context and enhanced their clinical practice within primary care. Furthermore, the results inform educators on how to effectively employ primary care-related SBT. Overall, this study supports the need for an increased application of SBT within primary care.

**Trial registration:**

Not relevant.

**Supplementary Information:**

The online version contains supplementary material available at 10.1186/s12912-024-01886-0.

## Background

Perceived as the gold standard of experiential learning, clinical placement comprises a fundamental component of nursing education. This interaction with the real-life workplace provides nursing students with the opportunity to apply their theoretical knowledge to clinical practice, develop their professional identity and contemplate their future careers [1, 2]. However, despite its multifaceted benefits, learning within the clinical environment is not without its obstacles, with students challenged by lack of access to adequate supervision, suboptimal learning encounters and passive involvement in clinical tasks [3, 4]. Such experiences of clinical placement are problematic, given the demonstrated association between student satisfaction with clinical placement and learning outcomes, motivation to continue their nursing education and student well-being [2]. As such, approaches to enhance the educational experience during clinical placement rotations have been explored.

One such approach is the supplementation of clinical placement with simulation-based training (SBT), an alternative experiential learning method. With its ability to effectively train a multitude of learning outcomes across a variety of clinical scenarios, SBT has been widely applied within nursing education [5, 6]. SBT has previously been perceived to bridge the gap between theory and practice, allowing nursing students to prepare for entering the clinical environment. However, given the challenges of learning in the clinical environment, studies have begun exploring the ability of SBT to replace clinical placement. In this context, SBT has been identified to provide a more concentrated and efficient educational approach, with the opportunity for students to undertake activities at higher levels of functioning [7]. Furthermore, combining clinical placements with SBT appears to increase participants’ knowledge acquisition and fulfillment of learning needs, compared to clinical placements alone [8].

Although these findings suggest the need for a new constellation for clinical placements, there is a lack of research exploring the advantages and disadvantages of this approach across different clinical contexts [9, 10]. Hence, the ways in which SBT can supplement clinical placement across different settings are currently unknown. This is particularly the case for primary care education, given the general underutilisation of SBT in this context, with simulation scenarios often taking place within a hospital setting [11]. Moreover, when adopted within primary care, SBT is typically employed to train communication skills using standardised patients, and hence, the current literature has not explored the breadth of clinical tasks performed in this context [12]. Further research is therefore required to understand the adaption and possible applications of SBT in a primary care setting.

To address these gaps in the literature, this qualitative study aimed to explore the perceived educational value of a supplemental simulation-based course for nursing students on clinical placement in primary care. Moreover, a secondary aim of this study was to outline how SBT can be adapted to a primary care context. The findings are intended to offer an insight into the potential advantages and disadvantages of combining clinical placement and SBT in primary care, as well as inform educators on how to effectively employ SBT within this context.

## Method

This is a qualitative descriptive study, conducted at the Copenhagen Academy for Medical Education and Simulation, Denmark, between September 2022 and July 2023.

### Sample

All thirty-five sixth-semester nursing students, enrolled at University College Copenhagen, and currently on placement in primary care within the Municipality of Copenhagen, were invited to participate. A convenience sampling technique was adopted, whereby participants were selected based on the location of their clinical placement in primary care. Although primary care in Denmark covers all healthcare provided outside of a hospital setting, e.g. general practice, homecare and nursing homes, nursing students are only placed within a nursing home and homecare setting during their primary care clinical placement. Clinical placements comprised 40 h per week for 20 weeks.

### Intervention

**Fig. 1:**
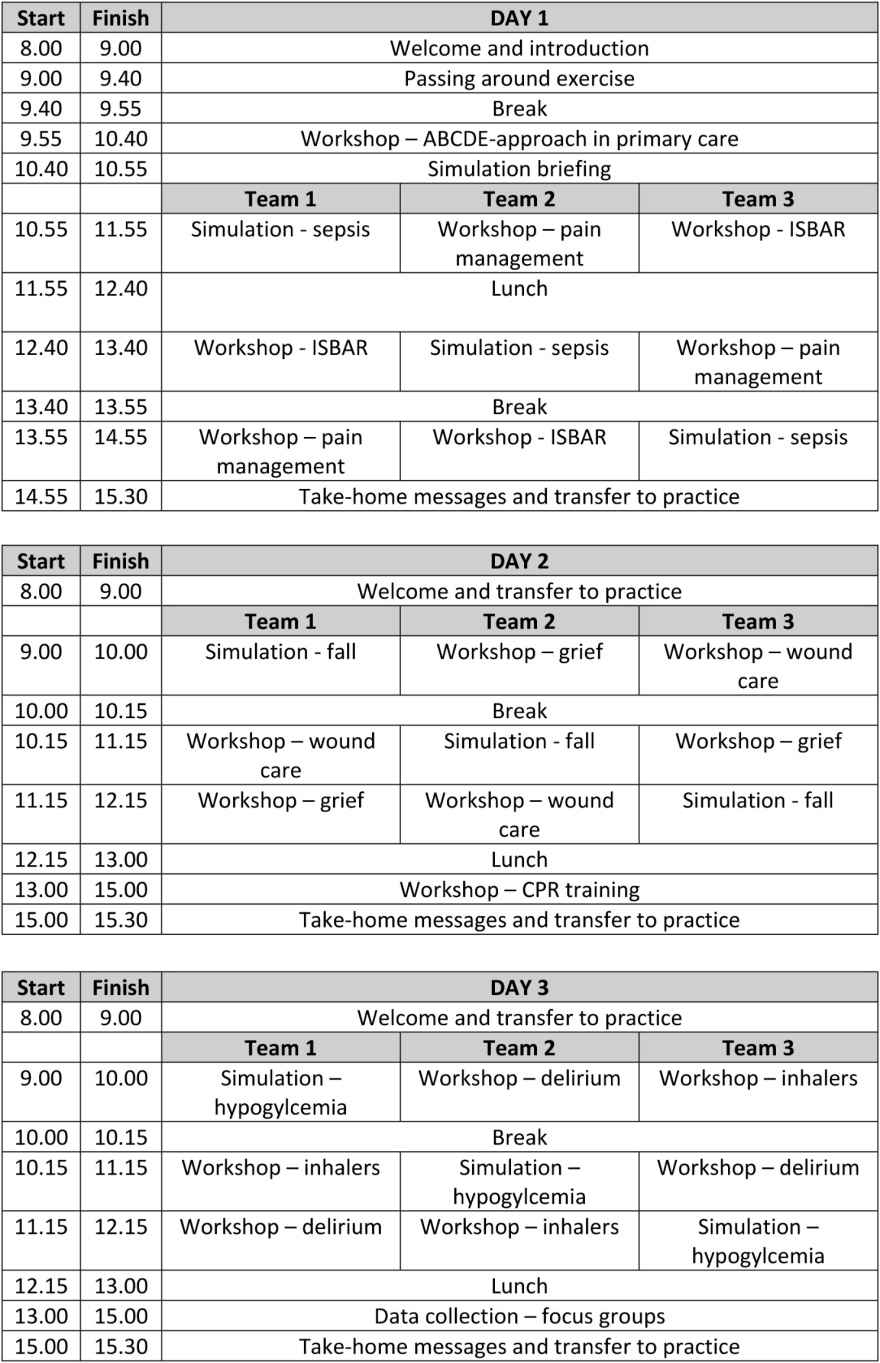
Program of the intervention

The intervention comprised a three-day simulation course covering core nursing competencies and common clinical conditions encountered within primary care. It was provided as a replacement for normal clinical activities, as it had been observed that previous cohorts of nursing students found the clinical placement in primary care overwhelming. The intervention was intended to support participants in their transition to primary care, encouraging their active participation in clinical activities and enhancing the overall educational impact of the clinical placement. The program is outlined in Fig. 1, whilst a more detailed description of each session is provided in supplementary file 1. The intervention was developed in collaboration with the Municipality of Copenhagen, and the topics taught were based on their insight into the participants’ challenges during placement.

The three days were divided over the course of three months, with participants attending one day a month for three months. Participants were often divided into small groups of approximately five, with the aim of actively involving all participants within the sessions. All teaching was conducted by nurse educators, who had previously completed a three-day simulation facilitation course and had previous experience in facilitating simulation training. Nurses were provided with detailed lesson plans for each teaching session, outlining learning objectives, educational activities and timeframes of the individual activities. Scenarios and scripts were also provided for all simulation training activities.

Teaching sessions were divided into traditional simulation training and workshops with integrated simulation training. Both forms of simulation were conducted using standardised patients, and in all scenarios, participants were functioning as qualified nurses. The traditional simulation training sessions were conducted in a simulation room, decorated as a patient’s living room, and the standardised patient was played by a medical student, trained in simulating patients, with the clinical findings being verbally provided by the facilitator. In contrast, workshops with integrated simulation training were conducted within a classroom and included both a presentation of theoretical content and a short simulation exercise. In this case, the standardised patient was played by the nurse educator. A detailed description of both forms of simulation training are provided in Table 1, outlining how these sessions were conducted. Both debriefing and feedback sessions were conducted immediately after the completion of simulation scenarios. Debriefing applied Steinwach’s model for facilitating debriefing, whilst feedback utilised Pendelton’s principles for feedback [13, 14].

**Table 1:** A description of traditional simulation training and workshops with intergrated simulation training

**Description of a traditional simulation training session– Sepsis**
**Learning objectives**: To be able to: • Apply the principles of ABCDE to the assessment and management of acutely unwell patients in a primary care setting • Interpret clinical findings to calculate a patient’s TOBS score and identify the appropriate management in accordance with the score • Demonstrate social and cognitive skills during an acute situation **Simulation approach**: Standardised patient, simulated by a medical student, who provided the history. The facilitator provided the findings of the physical examination and played the role of the contact person, when prompted. **Division of roles**: Two students completed the scenario, whilst the remaining students observed. Observers were given specific roles, according to the learning objectives. **Brief**: Pia/Per is an 81-year-old female/male, who lives alone in their own home/nursing home. You are visiting Pia/Per to change their dressing. They are known with hypertension and atrial fibrillation. They are immobile and use a wheelchair. **Scenario**: Pia/Per is an 81-year-old female/male, who lives alone in their own home/nursing home. The patient has a chronic wound, and today, the dressing needs to be changed. The scenario begins with two nursing students visiting the patient. When they enter, the patient is lying in bed, quiet, and breathing rapidly. The patient cannot answer questions clearly, and instead complains of stomach pain and feeling cold. The patient appears confused, which is unusual for them. The wound is clean and healing. Students must systematically assess the patient, initiate initial management and contact relevant health professionals for help **Debrief**: The debrief is divided into four phases: set the scene, description phase, analysis phase and application phase. The facilitator sets the scene, describing the purpose and structure of the debrief. Students describe the scenario and how the participants approached the patient, ensuring a shared perception of what happened. Students analyse the scenario, discussing what went well, which challenges occurred and what could have been done differently. Students discuss what they have learned from the simulation/debriefing and how they will apply their learning to their clinical practice. **Time**: 50 min divided: 5 min brief, 20 min scenario and 25 min debrief. **Equipment**: Simulation room decorated as a patients home/nursing home, telephone, the patient’s usual medications, rucksack carried by nurses in primary care containing the basic equipment required to assess and manage acutely unwell patients.

**Description of a workshop with intergrated simulation training session– ISBAR**
**Learning objectives**: To be able to: • Explain the importance of ISBAR in communication with others • Describe the five steps of ISBAR • Apply ISBAR to ensure safe and effective communication with others **Teaching plan**: The session starts with the facilitator reviewing the principles of ISBAR and its five components, concluding with the facilitator providing a demonstration. Afterwards, all students must individually prepare an ISBAR from a patient case provided, including identifying who they would contact. Using their prepared ISBAR, each student will then complete a simulation scenario, with the facilitator acting as the contact person. The facilitator sits behind a screen to simulate a telephone conversation. The other students observe the interaction and provide feedback, along with the facilitator. The session concludes with a summary of the key learning points. **Simulation approach**: Standardised contact person, played by the facilitator. **Division of roles**: Students took turns participating in the scenario, ensuring all students had the opportunity to practice ISBAR and receive feedback, as well as giving feedback. **Brief**: Before ringing for help, prepare ISBAR based on the patient’s situation and vital signs and consider who you will ring for help. **Example scenario**: Niels is a 66-year-old male, who is known with type 1 diabetes. His diabetes has been poorly regulated for a long time, and when you arrive to review Niel’s foot ulcer, he is confused and sweating. Vital signs are: A- Clear airway B- RF 18, Sats 98%, no cyanosis, no crepitations C- Pulse 92 bpm, regular, BP 168/92, CR < 2s, sweating D- GCS 15, equal and reactive pupils, BS 3.1mmol/L E- Temp 38.9, no abdominal tenderness **Feedback model**: The feedback comprised four steps: the student describes what went well, followed by comments from the observers and facilitator, and then the student describes what could have been improved, followed by comments from the observers and facilitator. **Time**: 50 min divided: 10 min to review principles of ISBAR, 7 min per student to practice ISBAR and receive feedback from peers/facilitator, 5 min to summarise key points. **Equipment**: Chairs, dividing screen, ISBAR preparation template, five patient cases, whiteboard.

### Data collection

Data was collected through four focus group interviews, conducted immediately after the completion of day three of the intervention. The focus groups were conducted separately from all educational activities. The interviews lasted 40–58 min, were audio recorded and had a mean group size of eight. Two interviewers, experienced in qualitative research methods, conducted the interviews together. The interviewers were not educators on the course and had not previously been involved in running the course. A semi-structured interview guide was used (supplementary file 2), focusing on the participants’ perceptions and the educational impact of the intervention.

### Data analysis

Data was electronically transcribed verbatim using Whisper (OpenAI model) and manually checked for accuracy (LB). Data was analysed using inductive thematic analysis (LB), as outlined by Braun & Clarke [15]. As the thematic analysis adopted an inductive approach, the themes were established through the data itself, instead of approaching the data with a preexisting framework. The process comprised six steps: (1) establishing familiarity with the data by reading the data and identifying initial ideas, (2) generating codes by assigning short phrases to segments of data, (3) generating themes by identifying patterns within codes and arranging them into meaningful groups, (4) reviewing themes by assessing whether the themes are representative of the dataset and (5) defining themes by naming and formulating what each theme represents. Data saturation was confirmed at both steps two and three, confirming that sufficient data had been collected to address the research aims. Each step of the analysis was validated by DØ, through both a review of the process and a discussion of the findings. The analysis was manually conducted.

## Results

A total of 31 nursing students participated in this study. Thematic analysis revealed seven themes and example citations are provided for each of these in Table 2.

**Table 2:** Example citations underlying the themes and subthemes

Themes	Example citation
Perceptions	*“I think it is really good to have this course, which is related to homecare. I think it has been clear throughout the teaching that it makes sense for us. It would have been something else, if we came to a course, where the simulations were in a hospital department. We would think, what could I use this for right now?”*
	*“I think the course has really made sense in relation to where we are in our education now, specifically primary care.… There have been many things we have experienced, which has increased our level when we are our there [clinical placement], as we have simulated these situations.”*
Educational value	*“But the thing about what you actually have to keep an eye on and write, that’s a big part of my clinical placement. And I have been able to see a difference in how I did it before I had been on the course, compared to what I look at and what I write when I make observations now. And it is as if you are looking at two completely different things. In one it says, yes, the treatment plan is followed. And in the second one, it says everything about wound edge, etc. So I took that with me. And then just in experience. Learn to use the clinical tools.”*
	*“I’ve been able to use the teaching about wounds, but otherwise it’s more in a way that… I haven’t encountered any acute situations yet, but I know that if I had to, I feel prepared for it. And it has helped a lot that I know which tools I can use in such a situation, should it happen. And I actually don’t feel nervous or afraid to come out to anyone. So, it has been extremely giving also in relation to the fact that I am no longer nervous to drive out alone to a patient.”*
Simulation adjustments to primary care	*“I also want a simulation with cardiopulmonary resuscitation, where they have also placed the patient up a plant and under a chair, and you also have to pull them out from that, because you don’t always find them lying on the ground, ready… and someone who was in the bath and who you have to dry, and things like that to remember.”*
	*“For example, when we are simulating, it is important to know what the time is. Because after 4 o’clock it is 1813 [we should ring to].”*
Educators’ competencies	*“You learn so much more, when you are with somebody, who is a wound care nurse, who actually teaches you that it is these things, you should look for. And this is how it looked, what you should do.”*
	*“They [educators] are some good role models with broad clinical experience. They exhibit confidence in what works and the teaching make sense. ”*
Learning needs within primary care	*“It would be really smart to have something like this interprofessional teamwork across sectors, something like, how does the collaboration work. Yes, some teamwork things like flowcharts of the communication agreement, what exactly should we do, what should we expect, what we will get from the hospital, what should patients take with them when they are admitted, how is the contact with the outpatient clinic, who is it, just like that command-line.”*
	*“I thought a subject could maybe be something like medication processes.… You could prepare some medications and in that way also administer, try calculating mediation doses.”*
Challenges of clinical placement	*“And the great thing about training as a nurse is that we are on clinical placement a lot, but there are also some, I hear, who say… they’ve never done stoma care, never tried NEWS-scoring before, never been in an emergency situation and had to call for help. So in a way, yes, having more simulations, where you are really focused on a topic, I think would be good to include more in nursing education.”*
	*“I think it’s great that we are so mixed and many, and to hear each other’s experiences from clinical placement.… We are learning from the other students too.”*
Career guidance	*“I’ve been quite inspired for what I’d like to do afterwards. I have been introduced to some topics and I think it has been really exciting. And have been asked about my direction for what I might like to do, and have talked to the teachers, where they have been good and guided me in how to reach my goal.”*
	*“We talk a lot about dropout and people who don’t want to be in this profession anymore and I think it has been great to meet some educators, who have been really happy with their jobs. The coolest job in the world.… And I think it’s been really encouraging or motivating. I think they have motivated us really well to think about the fact that it is actually a really good field.”*

### Perceptions

Participants’ responses to the intervention’s focus on primary care were overwhelmingly positive, given the alignment with their current clinical placement. Moreover, the selected topics were described as addressing many of the key aspects of working within this context. The relevance of the content to primary care was engaging and motivating, as participants could immediately apply their newly acquired competencies during clinical placement. Participants indicated that scenarios in a hospital setting would not have achieved this effect, as learning could not be transferred to its clinical context.

### Educational value

Participants praised the educational value of the intervention, stating that they were more confident in their clinical practice of the topics taught and had a greater understanding of clinical contexts and the rationale underlying decision-making in primary care. Participants were also better able to understand and interpret patient’s electronic health records, as they were able to define a greater range of clinical terms adopted in primary care. Moreover, participants felt more prepared for situations that are not often encountered in primary care, such as cardiac arrest, as they had acquired experience in applying clinical tools such as Identification, Situation, Background, Assessment and Recommendation (ISBAR), Airway, Breathing, Circulation, Disability and Exposure (ACBDE) and Cardiopulmonary Resuscitation (CPR) algorithm. As a result, they were no longer anxious about which situations they may encounter in an out-of-hospital context. Specific areas in which participants had applied their learning in practice included wound care, pain management, managing grief reactions, communicating with colleagues using ISBAR, clinical decision-making and documenting the essential information following a patient encounter. Generally, their participation in the intervention had increased participants’ ability to undertake tasks independently whilst on clinical placement and adopt a systematic approach to patient contacts.

### Simulation adjustments to primary care

Participants provided many valuable insights into the necessary adaptation of simulation training to a primary care setting. A fundamental insight was the simulation equipment used to convey vital signs and examination findings. As continuous monitoring is not available in primary care, the facilitator provided this information. However, participants indicated that this negatively affected the fidelity of the scenario and the provision of information through the equipment employed in primary care would have been advantageous. A significant factor was access to training IT-systems, providing access to the patient’s electronic health record and clinical guidelines. This would have allowed participants to orientate themselves about the patient beforehand and employ clinical support tools during the scenario, reflecting the realities of clinical practice. Furthermore, participants indicated that scenario design should incorporate potential barriers unique to primary care. For example, in acute situations, patients can be found in difficult-to-reach positions, presenting a barrier to providing the necessary care, which participants are less familiar with. As such, standardised patients or manikins should not always be lying in bed awaiting help, but instead be presented in ways that can be encountered in primary care. Alternatively, organisational barriers can also be experienced, such as the need to discuss specific examination procedures with a GP before their employment and these elements could be incorporated into scenarios. Other insights addressed the practical aspect of working within primary care, such as the importance of providing the time of the scenario to indicate which sources of help are available, i.e. GP, the acute team or out-of-hours telephone.

As nursing students can be placed in homecare or a nursing home during their clinical placement, the setting of the simulation scenario during the intervention depended on where the group was primarily based. However, participants highlighted the significant difference between the roles and responsibilities of nurses across these contexts, whereby homecare is primarily nursing-related, whereas nursing homes can include a mixture of nurse-related and healthcare assistant-related tasks. This difference was identified as a necessary consideration in the planning of simulation scenarios, highlighting the need to consider the significant variation across primary care settings.

### Educators’ competencies

Educators were described as engaged, motivational, competent and role models. This was primarily based on the educators’ broad clinical experience, which they were able to integrate into their teaching, providing clinical context to theoretical content. As a result, educators were able to provide realistic assessments of what works in clinical practice and were perceived as experts in the topics being taught. On a personal level, educators were also described as being good at praising participants and expressed positive perceptions regarding the career of a nurse. Furthermore, the continuity of two educators throughout the intervention increased the participants’ psychological safety and created a personalised learning experience, whereby educators could relate content to participants’ individual learning needs.

### Learning needs within primary care

Participants suggested an array of topics that would be valuable to train using simulation. This included the assessment and management of a range of clinical conditions, including sepsis, dehydration, urinary tract infections, dementia, depression, diabetes and opioid misuse. Moreover, participants identified a range of procedural skills that could be trained in this context such as preparing and administering medication, cannulation, catheterisation, stoma care, compression therapy, measuring blood sugar and inserting nasogastric tubes. However, participants also outlined potential topics that address the broader nursing roles. These included interprofessional teamwork across primary and secondary care, such as communication between sectors, preparing a patient for admission and receiving a patient after discharge, as well as interprofessional teamwork with the range of stakeholders within primary care. Broader topics also included professionalism and resilience, particularly regarding how to handle the feelings, thoughts and emotions that arise from challenging clinical encounters.

### Challenges of clinical placement

Participants identified a range of challenges of their clinical placement within primary care for which the intervention compensated. Participants experienced a lack of clinical supervision whilst on placement, with supervisors not actively engaging participants in discussions about clinical care and participants being allocated tasks without sufficient training. Numerous contributing factors were highlighted, with supervisors lacking time, insight into participants’ learning needs and updated clinical knowledge. Moreover, participants recognised the opportunistic nature of learning whilst on clinical placement, with participants particularly highlighting the lack of exposure to acute situations. Generally, the wide variance in clinical situations encountered across the different placement sites in primary care was deemed to significantly impact the learning opportunities encountered. The isolated nature of primary care was recognised as an important factor here, as the sparse contact with their peers limited the participants’ opportunity to learn from each other’s experiences and understand the nursing role across the spectrum of primary care.

### Career guidance

Participants indicated that the intervention had improved their perception of working within primary care, with many inspired to consider a future career within this setting. Overall, the intervention demonstrated the variety available within nursing, which provided inspiration for future career pathways. Moreover, the enthusiasm for nursing exhibited by educators offered a welcome contrast to the typically negative discussion surrounding the profession, motivating participants to continue their training at a time when they were doubting their career choice.

## Discussion

This study explored the educational value of a supplementary simulation-based course for nursing students on clinical placement in primary care, as well as the adaptation of SBT to this context. The results revealed seven themes, addressing participants’ perceptions of the intervention, their learning needs with primary care and the adjustment of SBT to this context. Overall, participant responses to the intervention were overwhelmingly positive, appreciating its ability to fulfill their learning needs and enhance their experience of clinical placement in primary care. Participants encouraged an increased application of primary care-related SBT, outlining numerous advantages of such an approach, and valuable insights into how to further adapt SBT within this context were offered.

Regarding the educational value of the intervention, the findings indicate that participants achieved both knowledge gain and skill acquisition, with learning also being transferred to clinical practice. Much of the knowledge gain related to participants’ comprehension of clinical situations and interpretation of patient’s electronic health records. There was also evidence of knowledge gain relating to the management of specific conditions, such as wound care and pain management. These findings suggest participants acquired the fundamental knowledge underpinning clinical practice in primary care; an essential educational gain given the inadequate preparation of nursing students for working within this context identified in the literature [16, 17]. Moreover, skill acquisition primarily related to essential clinical tools such as the ACBDE assessment of patients, the CPR-algorithm and communication using ISBAR. Mastery of these tools allowed participants to feel more prepared for clinical encounters and enabled participants to undertake tasks independently. The increased autonomy of participants represents an important educational impact within the context of primary care, given the autonomous nature of the work within this context [18]. As such, the intervention appears to offer important educational benefits, supporting the effectiveness of SBT within primary care. Generally, there is a paucity of studies exploring the value of SBT for nursing students within this context, with the application of SBT to primary care representing a growing area of interest [12, 19]. Currently available studies appear to align with the findings of this study, indicating that SBT is of similar value in primary care, as in secondary care [19–21].

In addition to the educational impact of the intervention, the results also identified positive effects on wider issues, such as participant well-being, retention to nursing education and career guidance. Their experiences of clinical placement in primary care, combined with the negative discussions surrounding the nursing profession encounter in this context, had left participants feeling disengaged with their education and questioning their career choice. Moreover, the solitary nature of primary care limited their understanding of the variation and opportunities across primary care. However, the range of learning opportunities encountered through the intervention and the engagement exhibited by the educators appeared to have supported participants in adjusting to clinical placement in primary care and motivated them to continue their studies. Furthermore, participants were more likely to consider a career in primary care, particularly as they felt more informed of the career options available within this context. Previous studies have identified that the acute-focused nursing curriculum leaves nursing students inadequately prepared for working within a primary care context [16, 17]. The resulting negative clinical experiences can have lasting consequences for a nurse’s career choice, particularly given the pre-existing negative perceptions of working within primary care identified within the literature [17, 22, 23]. However, the rising demands, workforce shortages and recruitment challenges impacting this context, highlight the need for increased focus on the transition to primary care and ongoing support during clinical placements [23]. An increased application of primary care-related SBT offers a potential solution to these issues and supports the need for increased employment of this approach.

As well as the educational advantages supporting an increased application of primary care-related SBT, the findings also highlight the importance of adapting simulations to this context. Participants particularly appreciated the alignment of the intervention to their current clinical placement, indicating that hospital-based simulations would not have achieved an equal educational impact. This alignment allowed participants to recognise the relevance of the content and directly apply their learning to clinical practice to achieve a greater level of active involvement during clinical placement. These findings support the notion that SBT is context-depending and previous training through hospital-based simulations does not provide a sufficient replacement for primary care-focused SBT [12, 24]. Moreover, the provision of primary care-related SBT can also serve to expand the participants’ clinical experience within this context. Nurses opting to work in primary care encounter various employment opportunities, including general practice, nursing homes and homecare. However, during clinical placement nursing students are only allocated to one such facility, limiting the breadth of their experience within this context and adversely affecting their understanding of the organisation of primary care. As such, SBT can compensate for these challenges by training scenarios across the different settings and providing participants with broader clinical experience within primary care.

In many ways, the design and facilitation of SBT within primary care is similar to secondary care. The pre-brief, debrief and facilitation did not require specific adaptations beyond following the standards of Healthcare Simulation Standards of Best Practice [25]. Instead, many of the adaptations related to simulation design, particularly the criteria related to designing the scenario, creating the perception of reality and planning a learner-centred approach [26]. Numerous adjustments were made to the simulations to ensure they aligned with a primary care setting and created an acceptable level of realism. For example, the simulation room was decorated as the patient’s bedroom, representing either their own bedroom or that of a nursing home. All indicators of a hospital setting were removed or covered and the standardised patient was dressed in their own clothes. Additionally, the scenarios were designed to represent the typical scenarios encountered within primary care and the actions expected to be undertaken by the participants were adjusted for this setting. For example, low oxygen saturation could only be managed by optimising the position of the patient rather than giving oxygen. This aligned with the equipment available during the scenario, with participants only provided with the equipment they have available within a primary care context. Finally, all documents provided to participants during the scenario adopted the same format and level of detail as the documents encountered in practice. However, SBT within a primary care setting is a new area of development and there are limited examples of similar interventions within the literature [12, 19]. Beyond the adjustments made by the educational team, valuable insights into the implementation of SBT in a primary care context were provided by participants. These related to both potential simulation scenarios and learning objectives, as well as factors related to the design of the simulation. Such insights offer important information on how to effectively expand the application of SBT in primary care to fulfill students’ learning needs and increase the fidelity of such activities to enhance the educational experience.

## Limitations

The qualitative study was conducted at a single centre, limiting the transferability of the results. As data was collected using self-reporting outcomes, there is a risk of response bias influencing the results, including the tendency to overestimate the educational value of the intervention and overreport positive outcomes. This could potentially have skewed the results towards a more favourable depiction of the intervention, minimising potential disadvantages or challenges of the supplementation of clinical placement with SBT. Finally, the researchers conducting this qualitative study were also involved in the development and planning of the intervention. This involvement could have influenced the analysis of the findings towards a more positive evaluation of the simulation-based course.

## Conclusion

This qualitative study explored the perceived educational value of a supplemental simulation-based course for nursing students on clinical placement in primary care, as well as the adaptation of SBT to this context. Participants’ responses to the intervention were overwhelmingly positively, as they appreciated its relevance to their current clinical placement, its ability to fulfill their learning needs and the subsequent enhancement of their experience of clinical placement. Overall, participants felt more prepared for the situations they could encounter within primary care and participants were able to directly apply their learning from the intervention to their clinical practice. Additionally, important insights regarding the adaption of SBT to a primary care context were identified, as well as potential scenarios that could be trained using SBT, with the findings supporting an increased application of this training method within primary care.

### Electronic supplementary material

Below is the link to the electronic supplementary material.

Supplementary Material 1

## Data Availability

The datasets used and/or analysed during the current study are available from the corresponding author on reasonable request.
