# Supplementary material for: A qualitative study of the value of simulation-based training for nursing students in primary care
Source: BMC Nurs. 2024 May 6;23:308. doi: 10.1186/s12912-024-01886-0 (PMC11071186; doi:10.1186/s12912-024-01886-0)
Supplement: Supplementary file 1 — Supplementary Material 1 [file 12912_2024_1886_MOESM1_ESM.docx]

**Supplementary File 1.** Detailed description of each session of the intervention

**Day 1**

*Welcome and introduction*

Facilitators introduce themselves and the course, followed by a presentation of social and cognitive skills (non-technical skills), i.e., what social and cognitive skills are, why they are important, and how they are applied in clinical practice. The session aims to:

- Introduce the students to the instructors and provide an overview of the course
- Describe the different social and cognitive skills and their importance in nursing

*Workshop - ABCDE-approach in primary care*

A discussion of the signs/symptoms and possible interventions at each step of the ABCDE-approach, followed by a demonstration by the facilitators. Afterwards, students calculate the TOBS-score of multiple patient cases and identify the appropriate actions to take according to the score. The session aims to:

- Increase students' understanding of when and how to apply the ABCDE-approach
- Provide students with the necessary knowledge and skills required to undertake an ABCDE-assessment
- Train students in calculating the TOBS-score of acutely unwell patients and identifying the appropriate actions to take according to the score

*Workshop - Pain management*

A discussion of the different types of pain, assessment of a patient with pain and approaches to pain management. Afterwards, students train their pain assessment skills, with the facilitator playing a patient with pain. The session aims to:

- Provide students with knowledge of the different types of pain and their management
- Provide students with knowledge and skills in pain assessment

*Workshop - ISBAR*

A discussion of the steps involved in ISBAR-communication. Afterwards, students prepare an ISBAR-referral from patient cases and trains their ISBAR-communication with the facilitator plays the role of the receiving health professional. The session aims to:

- Ensure that students posses the necessary knowledge and skills required to utilize the ISBAR approach in their communication with other health professionals

*Simulation - Sepsis*

Pia/Per is an 81-year-old female/male, who lives alone in their own home/nursing home. The patient has a chronic wound, and today, the dressing needs to be changed. The scenario begins with two nursing students visiting the patient to change their dressing. When they enter, the patient is lying in bed, quiet, and breathing rapidly. The patient cannot answer questions clearly, and instead complains of stomach pain and feeling cold. The patient appears confused, which is unusual for them. The wound is clean and healing. Students must systematically assess the patient, initiate initial management and contact relevant health professionals for help. The session aims to:

- Provide students with the opportunity to train the ABCDE-assessment of acutely unwell patients and ISBAR-communication
- Increase students’ awareness of situational awareness and decision-making in acute scenarios

*Take-home messages and transfer to practice*

Facilitators summarize key points from the sessions and students reflect upon what they have learnt and how they can apply their learning into clinical practice.

**Day 2**

*Welcome and transfer to practice*

Facilitators introduce the second day and students reflect on the previous day, ask any questions that have arisen since and discuss how they have applied their learning into clinical practice.

*Workshop - Grief*

A presentation on grief, including normal grief reactions, complicated grief and how health professionals can support patients during the grief process and identify those in need for further treatment. The session aims to:

- Provide students with an understanding of the grief process
- Provide students with the tools necessary to support patients during the grief and identify patients with complicated grief

*Workshop - Wound care*

A discussion of the assessment of wounds, including the wound assessment triangle, and guidelines for the management of different types of wounds. Students also reviewed patient cases, with the task of describing the wound and identify the appropriate management. The session aims to:

- Provide students with the knowledge and skills required to undertake a holistic assessment of wounds
- Increase students’ knowledge of different methods and treatment options used in wound care

*Simulation - Fall*

Anne/Anders is an 82-year-old female/male, who lives alone in their own home/nursing home. The patient has a catheter, which needs to be changed. The patient has dementia, which has deteriorated since they lost their spouse last year. The scenario begins with two nursing students visiting the patient to change their catheter. When they enter, the patient is lying on the floor in severe pain and calling for help. Their right leg appears shortened and externally rotated and they have an abrasion on their forehead. Students must systematically assess the patient, initiate initial management and contact relevant health professionals for help. The session aims to:

- Provide students with the opportunity to train the ABCDE-assessment of acutely unwell patients and ISBAR-communication
- Increase students’ awareness of situational awareness and decision-making in acute scenarios

*Workshop - CPR training*

A discussion of the recognition of cardiac arrest and the steps of CPR in an out-of-hospital setting, combined with students practicing the different steps on manikins. The sessions aims to:

- Ensure students are able to recognize cardiac arrest and implement basic life support in an out-of-hospital setting.

*Take-home messages and transfer to practice*

Facilitators summarize key points from the sessions and students reflect upon what they have learnt and how they can apply their learning into clinical practice.

**Day 3**

*Welcome and transfer to practice*

Facilitators introduce the second day and students reflect on the previous day, ask any questions that have arisen since and discuss how they have applied their learning into clinical practice.

*Workshop - Delirium*

A case-based discussion that covers the cause, risk factors, identification and management of delirium. The session aims to:

- Provide students with knowledge about detection, prevention and management of delirium.

*Workshop - Inhalation medication*

A presentation of the different types of inhalators and a demonstration of their application. Afterwards, students practice informing patients of the correct use of the different inhalators, with the facilitator playing the patient. The session aims to:

- Increase the students' knowledge of different inhalation preparations and their application
- Train students in informing patients of the correct use of their inhalation medication

*Simulation - Hypoglycemia*

Greta/Peter is a 72-year-old female/male, who lives alone in their own home/nursing home. The patient has significantly impaired vision and is known with back pain, hypertension, and type 1 diabetes. The scenario begins with two nursing students visiting the patient, after they were discharged last night after a brief hospitalization due to severe back pain. The students find the patient with reduced consciousness and sluggish. They respond only briefly when spoken to. Students must systematically assess the patient, initiate initial management and contact relevant health professionals for help. The session aims to:

- Provide students with the opportunity to train the ABCDE-assessment of acutely unwell patients and ISBAR-communication
- Increase students’ awareness of situational awareness and decision-making in acute scenarios

*Take-home messages and transfer to practice*

Facilitators summarize key points from the sessions and students reflect upon what they have learnt and how they can apply their learning into clinical practice.

**Supplementary file 2.** Questions included in the interview guide

1. What do you think of the course?
2. For those who think the course has been good, why was the course particularly good?
3. For those who do not think the course was good, why was the course not good?
4. What do you think of the learning methods that were used during the course?
5. What did you get out of the course?
6. Have you applied your learning in the clinic, if so, how?
7. How will you continue to build upon what you have learned on the course?
8. Do you think that the course should be a permanent part of the primary care rotation within the Municipality of Copenhagen?
